# Control of Arms of Au Stars Size and its Dependent Cytotoxicity and Photosensitizer Effects in Photothermal Anticancer Therapy

**DOI:** 10.3390/ijms20205011

**Published:** 2019-10-10

**Authors:** Joanna Depciuch, Malgorzata Stec, Alexey Maximenko, Miroslawa Pawlyta, Jarek Baran, Magdalena Parlinska-Wojtan

**Affiliations:** 1 Institute of Nuclear Physics Polish Academy of Sciences, PL-31-342 Krakow, Poland; alexey.a.maximenko@gmail.com (A.M.); bpparlin@cyf-kr.edu.pl (M.P.-W.); 2 Department of Clinical Immunology, Institute of Pediatrics, Jagiellonian University Medical College, PL-30-663 Krakow, Poland; stecmalgorzata@gmail.com (M.S.); mibaran@cyf-kr.edu.pl (J.B.); 3 Institute of Engineering Materials and Biomaterials, Silesian University of Technology, Konarskiego 18A, 44100 Gliwice, Poland; miroslawa.pawlyta@polsl.pl

**Keywords:** gold nanostars, phototherapy, photosensitizer, cancer, star arms, synthesis mechanism

## Abstract

Gold nanostars (AuS NPs) are a very attractive nanomaterial, which is characterized by high effective transduction of the electromagnetic radiation into heat energy. Therefore, AuS NPs can be used as photosensitizers in photothermal therapy (PTT). However, understanding the photothermal conversion efficiency in nanostars is very important to select the most appropriate shape and size of AuS NPs. Therefore, in this article, the synthesis of AuS NPs with different lengths of star arms for potential application in PTT was investigated. Moreover, the formation mechanism of these AuS NPs depending on the reducer concentration is proposed. Transmission electron microscopy (TEM) with selected area diffraction (SEAD) and X-ray diffraction (X-Ray) showed that all the obtained AuS NPs are crystalline and have cores with similar values of the diagonal (parameter *d*), from 140 nm to 146 nm. However, the widths of the star arm edges (parameter *c*) and the lengths of the arms (parameter *a*) vary between 3.75 nm and 193 nm for AuS1 NPs to 6.25 nm and 356 nm for AuS4 NPs. Ultraviolet-visible (UV-Vis) spectra revealed that, with increasing edge widths and lengths of the star arms, the surface plasmon resonance (SPR) peak is shifted to the higher wavelengths, from 640 nm for AuS1 NPs to 770 nm for AuS4 NPs. Moreover, the increase of temperature in the AuS NPs solutions as well as the values of calculated photothermal efficiency grew with the elongation of the star arms. The potential application of AuS NPs in the PTT showed that the highest decrease of viability, around 75%, of cells cultured with AuS NPs and irradiated by lasers was noticed for AuS4 NPs with the longest arms, while the smallest changes were visible for gold nanostars with the shortest arms. The present study shows that photothermal properties of AuS NPs depend on edge widths and lengths of the star arms and the values of photothermal efficiency are higher with the increase of the arm lengths, which is correlated with the reducer concentration.

## 1. Introduction

Biocompatibility, chemical stability, and possibility of size and shape modification during synthesis are reasons that gold nanoparticles (Au NPs) are used in a multitude of fields, especially in medicine for virus detection [1], diagnostics [2], biosensing, drug carriers, and molecular imaging [3]. Furthermore, a large number of easily polarizable conduction electrons in Au NPs allows the interaction between nanoparticles and electromagnetic fields, which caused generation of nonlinear optical phenomena [4]. Moreover, in comparison with other chromophores, both organic and inorganic 2-nm Au NPs show a larger extinction cross section and a possibility of reaching 100% of light-to-heat conversion efficiency in the range of electromagnetic wavelengths used in photothermal anticancer therapy (PTT) [5].

The physical and chemical properties of Au NPs depend on the morphology (size and shape) and surface modification of the nanoparticles (ligands and used stabilizer) [6]. Also, the surface plasmon resonance (SPR) of the Au NPs is dependent on these features. The SPR is exploited in a wide range of gold nanoparticles applications. Oscillations of the surface electrons with a resonance to the incident frequency are caused by the electric field [7]. Propagation direction of the planar electric field and orientation of the nanostructures are responsible for the optical absorption and scattering in Au NPs, which are very important in PTT [8]. The plasmonic properties of gold nanoparticles are very well known for spherical Au NPs as well as Au nanorods. However, in comparison with these two kinds of shapes, AuS NPs have very interesting properties, which are dependent on their fancy shape [9,10]. Moreover, the sharp arms of the Au nanostars (AuS NPs) with a tunable, narrow band of SPR are responsible for the strong electromagnetic enhancement effects [11]. Localization of the electric field at the tip end causes strong dephasing of coherently oscillated surface electrons. This electron energy is transferred to the atomic lattice, effusing strong flux of heat at the metal−dielectric interface [12]. Moreover, high values of the ratio between absorption and scattering of light in AuS NPs are the reason that gold nanostars are one of the most effective agents for the transduction of photon energy into heat energy [13]. This causes AuS NPs to be used as effective photosensitizers in PTT. Moreover, the maximum plasmonic properties of AuS NPs and consequently the maximum thermal activity are localized in the arms of the stars; therefore, the morphology of these star shaped nanoparticles is crucial in the application of AuS NPs.

Taking into account the potential application of AuS NPs in medicine, the toxicity effect of these nanostructures needs to be explored [14,15]. Several studies showed that the toxicological properties of Au NPs depend on their size, shape, and surface chemistry [16,17]. Therefore, before applying the nanoparticles in medicine, the toxicity effect should be investigated. In this study, we determine the cytotoxicity effect of AuS NPs. Moreover, knowing that the thermal properties of AuS NPs depend on the morphology of star arms, we synthesized the gold nanostars with very similar sizes of nanoparticle cores and different lengths of star arms. Subsequently, we investigated the application of AuS NPs in the PTT. For this purpose, two lasers with 650-nm and 808-nm wavelengths were used to irradiate the SW480 and SW620 colon cell lines. Furthermore, in this study, we propose the synthesis mechanism of the AuS NPs dependent on the reducer concentration. For determining the physical properties of the AuS NPs, TEM with selected area diffraction (SEAD) patterns, XRD, and UV-Vis spectroscopy were used.

## 2. Results and Discussion

### 2.1. Morphology, Structure, and Optical Properties of Nanoparticles

**Figure 1:**
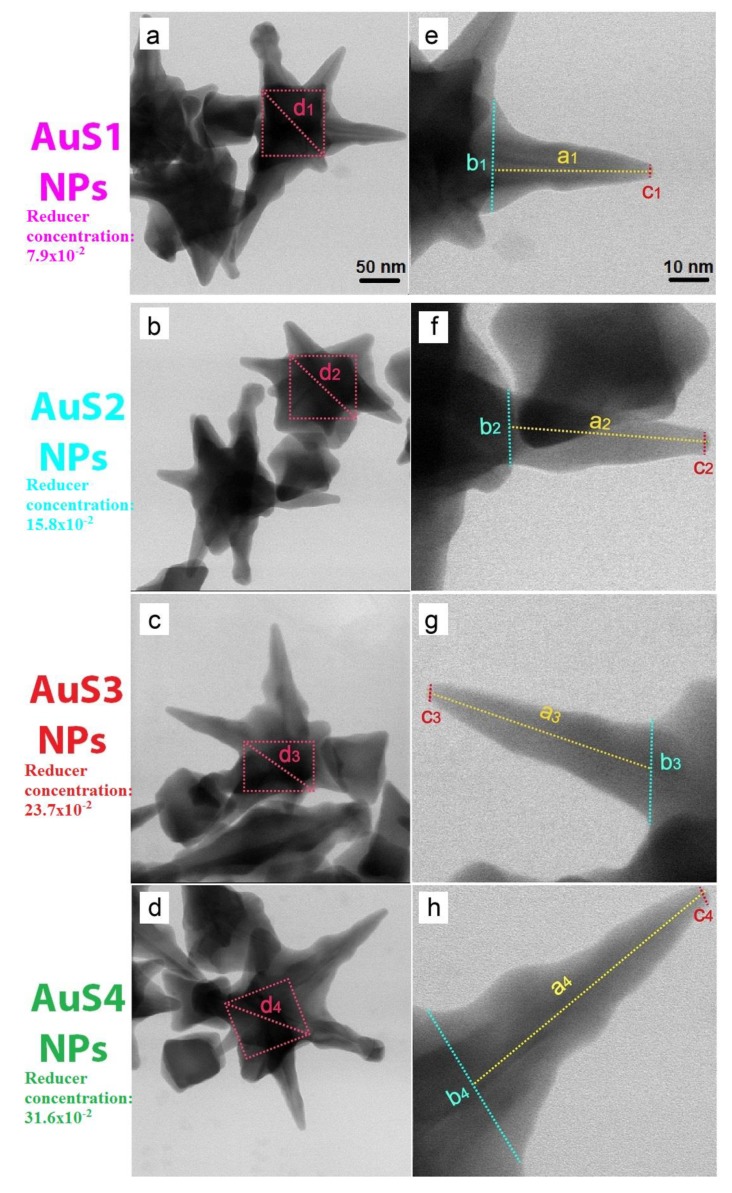
Scanning transmission electron microscopy (STEM) bright field (BF) overview images (**left column**) and magnified images (**right column**) of AuS NPs (gold nanostars): AuS1 NPs (**a**,**e**); AuS2 NPs (**b**,**f**); AuS3 NPs (**c**,**g**); and AuS4 NPs (**d**,**h**), with marked parameters presented in Table 1.

**Figure 2:**
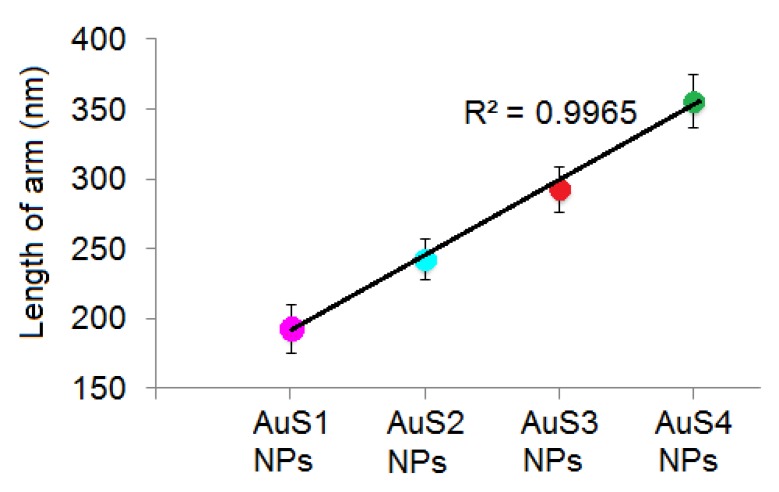
Dependence of the lengths of the star arms on the reducer concentration.

The morphology of the synthesized AuS NPs was analyzed by scanning transmission electron microscopy (STEM) using the bright field (BF) detector; see Figure 1. As expected, the changes in the reducer concentration had influence on the size of the gold nanostars as well as on the lengths of the star arms; see Figure 1a–h. Moreover, the STEM images showed that all four nanostars have a core in the center. These cores have a cube-like shape, and the diagonal size is similar in all obtained samples, from 140 nm to 146 nm. However, as we can see in Figure 1e–h, the lengths of nanostar arms (parameter a, marked by dashed yellow line) are different in all AuS NPs. Interestingly, the size of arms increases as a function of the reducer concentration; see Figure 2. For the smallest concentration of reducer, the arm length was around 193 nm, while for the highest concentration of the reducer, the arms were longer, reaching 356 nm. Moreover, also the lengths of the semimajor axis (b, c) are different and depend on the reducer concentration. However, in the case of the semimajor axis, we did not notice a linear dependence. All parameters for the four different AuS NPs, which are marked in Figure 1, are presented in Table 1.

**Figure 3:**
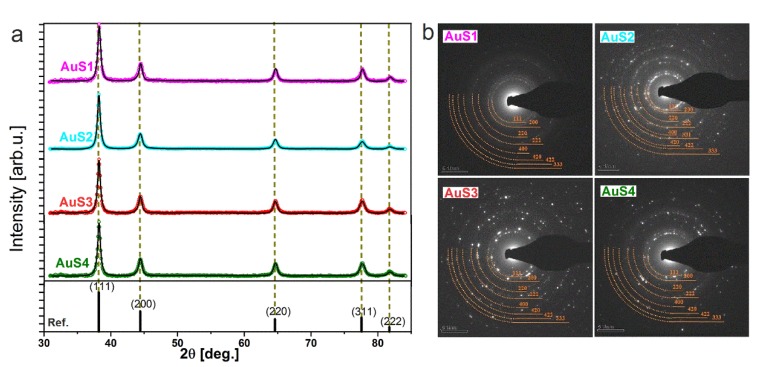
(**a**) X-ray diffraction patterns of AuS nanoparticles synthesized at different reducer concentrations, where open circles present experimental data and solid black lines correspond to calculated Rietveld refinement plots; (**b**) SAED patterns of the synthesized AuS NPs [19].

The global structure of the Au nanoparticles synthesized at different reducer concentrations was analyzed by X-ray diffraction and SAED patterns. As indicated by the dashed lines across Figure 3a, the experimental patterns for all synthesized AuS NPs exhibit peaks, which match the standard of gold, and a good refinement of the patterns was obtained by considering the gold fcc phase [18]. The calculated average coherent scattering lengths (<D>) for the nanoparticles, which we assumed to be equal to crystallite sizes, were around <D_Au_> ≈ 12 nm and did not change with the reducer concentration. We assume that the obtained values of the crystallite sizes originate from polycrystalline cores of AuS nanoparticles, of which the size remains unchanged with increases in the reducer concentration. On the other hand, we found that increasing the average length of the arm in the gold nanostars correlated with increasing the AuS NPs lattice parameter. Thus, the AuS4 NPs showed the largest lattice parameter, which was close to the bulk value of gold, 4.1547 Å; see Table 2 [19].

The SAED patterns were acquired with an aperture of 200 μm from the four analyzed nanoparticle samples (Figure 3b) and indexed with planes corresponding to the fcc structure of Au [20]. Although all SAED patterns consisted of rings, we observed differences in the respective patterns originating from the increasing lengths of the arms of Au nanostars in the samples from AuS1 NPs to AuS4 NPs. Indeed, the SAED pattern taken from the AuS1 NPs sample is composed from rather blurred rings, which results in the shortest arms (193.75 nm) of the stars. Indeed, the SEAD patterns of AuS3 NPs showed sharper rings and contained more and more spots compared to sample AuS2 NPs. As the arms become longer, the SAED pattern of sample AuS4 NPs exhibited sharper rings and contained the largest number of spots.

**Figure 4:**
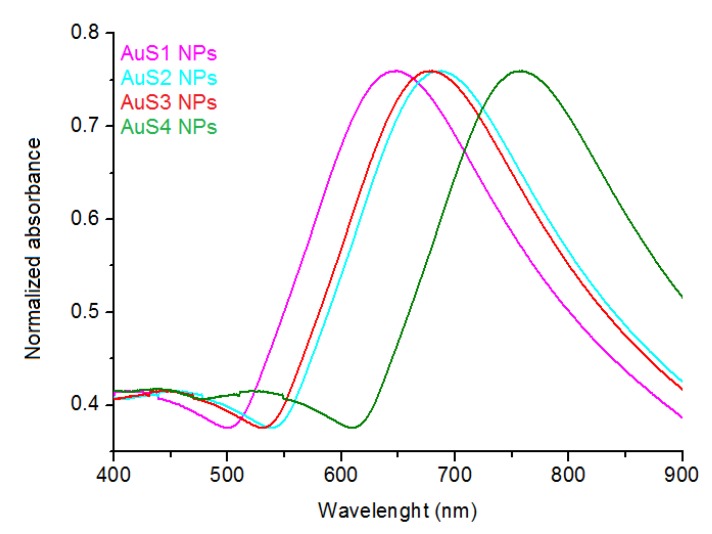
Surface plasmon absorptions of all synthesized gold nanostars.

The positions of the SPR peak, which plays an important role in the possibility of using the synthesized AuS NPs in PTT, are presented in Figure 4. The peak positions are between 640 nm for AuS1 NPs and 770 nm for AuS4 NPs. We can correlate the SPR peak maximum with the lengths of c semimajor axis. Along with the elongation of the c axis (Table 1), a shift of the peaks to higher wavelengths is observed. In PTT, it is very important that nanoparticles absorb light in wavelength ranges safe for healthy tissue. These ranges are between 650–850 nm or between 950–1350 nm [21]. As we can see, all synthesized AuS NPs absorb light in the biological range. Moreover, when we relate the UV-Vis spectra of AuS NPs with the length of their respective c axes, it is clearly visible that the positions of SPR peaks are dependent on the sharpness of the ends of the AuS NPs arms. Therefore, modification of the length of the c axis allows synthesizing nanoparticles with a specific position of the SPR peak in a controlled way.

### 2.2. Mechanism of Formation of AuS NPs with Control of Star Arms Lengths

**Figure 5:**
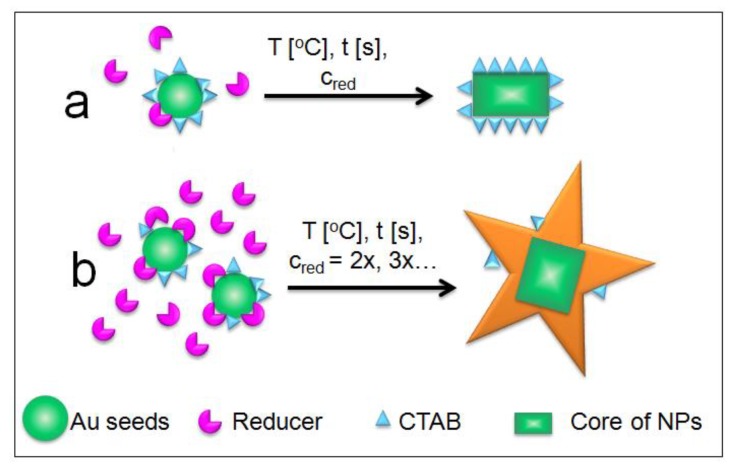
Scheme of the mechanism of gold nanostar formation with controlled lengths of star arms: with small concentration of reducer (**a**) and with high concentration of reducer (**b**). The scheme is not in scale.

Increasing the reducer concentration caused a decrease of time, which was needed for the reduction of gold(III) chloride trihydrate (HAuCl_4_). Moreover, higher reducer concentrations are responsible for the increase of the number of initial nucleation sites, which caused the obtained nanoparticles to have very similar shapes and sizes of the cores (Figure 1a–d), but they expanded in all directions, reaching a star shape. In addition, the higher number of nucleation sites caused the participation of each nucleation site to be smaller and nondominant, which also contributed to the formation of gold nanostars [22]. Moreover, cetrimonium bromide (CTAB), except preventing the agglomeration of nanoparticles, is also responsible for the initial stabilization of single gold nanoseeds, which, when coated with CTAB, grow in the {110} direction [23,24]. The above describe effects such as decrease of the HAuCl_4_ reduction time, increase of nucleation sites, decrease of the number of nanoparticles coated with CTAB, and nondominant direction of nanoparticle growth, which are caused by the increase of reducer concentration. A very similar effect was visible in the synthesis of spherical nanoparticles, e.g., silver nanoparticles (Ag NPs), where depending on the concentration of glucose, different sizes of Ag NPs were obtained [25]. Moreover, using different concentrations of reagents during the Au NPs synthesis, it is possible to obtain spherical Au NPs almost without any size distribution, which is very important in medical applications [26]. Therefore, we suspect that the mechanism of formation of gold nanostars with controlled star arms lengths is correlated with the addition of a defined amount of reducer. Thus, the scheme of this mechanism is presented in Figure 5.

### 2.3. Photothermal Conversion Efficiency of Obtained AuS NPs

**Figure 6:**
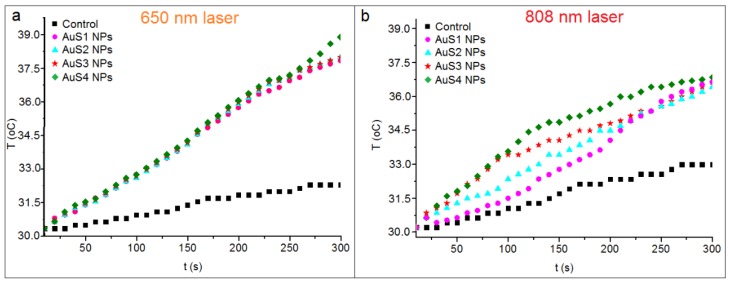
Changes of the temperature in AuS1 NP (pink circles); AuS2 NP (blue triangles); AuS3 NP (red stars), and AuS4 NP (green diamonds) solutions irradiated by 650-nm (**a**) and 808-nm (**b**) lasers. Black color corresponds to the water (control) solution.

The changes of temperature in the AuS1–AuS4 NP solutions, which were irradiated by 650-nm and 808-nm lasers, are shown in Figure 6. In comparison with the control water solution, the increases of temperature in the AuS1–AuS4 NP-containing solutions were observed for both laser wavelengths: 650 nm and 808 nm. When we compare the temperature increase, a higher increase for all nanoparticle solutions by about 1 °C was noticed for the 650-nm laser than for the 808-nm laser. Moreover, regardless of the used wavelengths, similar temperature increases in the all AuS NPs solutions were observed. The laser with the 650-nm wavelength caused an increase of the temperature of about ~8 °C after 5 min of irradiation, while for the 808-nm laser, the increase was about 7 °C. From the temperature increase data and using Equation (1), the photothermal efficiencies of the respective AuS NPs were calculated and are presented in Table 3.

Higher values of photothermal efficiency were observed for the 650-nm laser than for the 808-nm one. Moreover, for both wavelengths, the highest values of photothermal efficiency were found for the AuS4 NPs equal to 44% and 34%, respectively. However, the differences in the values of η between all synthesized AuS NPs were not high. The smallest value of η = 38% was observed for AuS1 NPs for the 650-nm laser, while for the 808-nm laser, the smallest value of photothermal efficiency = 32% was found for AuS2 NPs. Moreover, very similar values of the calculated parameter, for both 650-nm and 808-nm wavelengths, were observed for AuS2 NPs and AuS3 NPs. This could be related to the fact that the lengths of semimajor axes c, which define the sharpness of the end of the star arm, are similar in the AuS2 NPs and AuS3 NP samples, Table 1. The photothermal efficiency depends on the extinction coefficients, which determine the light absorption properties of substances [27]. This parameter can be calculated for each wavelength, e.g., 650 nm and 808 nm, like in this experiment. Chatterjee et al. showed that the highest values of extinction coefficients for wavelengths near to the biological range are for gold nanostars having the longest arms [13]. Consequently, these AuS NPs should have the highest values of photothermal efficiency. As we can see in Table 1, the longest semimajor axis c is in the AuS4 NPs. Also, for these AuS NPs, the increase of temperature after irradiation is the largest (Figure 6), and consequently, the values of photothermal efficiency reached the highest values. Moreover, when the semimajor axis c becomes shorter, the temperature after irradiation, as well as, the values of η decreased. Furthermore, Chatterjee et al. noted that the highest values of electric field intensity were also observed for nanoparticles with the smallest sharpness of the end of AuS NPs arms. Multiple sharp branches on the AuS NPs create a “lightning rod” effect, which enhances the local electromagnetic field dramatically [28]. This is the next reason why the AuS4 NPs with the highest values of parameter c (Table 1) had the most effective photothermal properties.

### 2.4. Simulation of PTT of Cancer Cells in the Presence of AuS NPs

**Figure 7:**
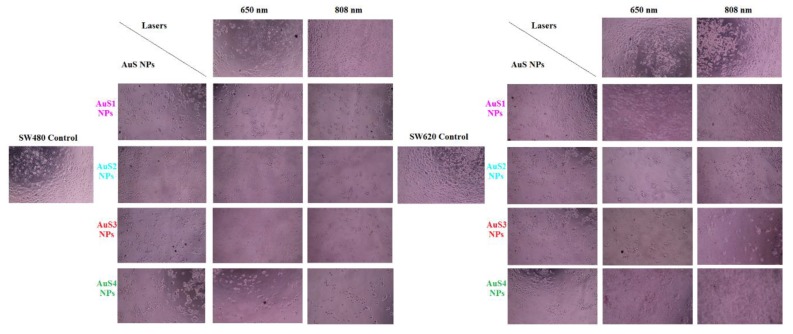
Light microscopy images of colon cancer cells (SW480 and SW620) showing their morphology: control, after addition of AuS1–AuS4 NPs and after irradiation with 650-nm and 808-nm lasers without and with AuS1-AuS4 NPs.

**Figure 8:**
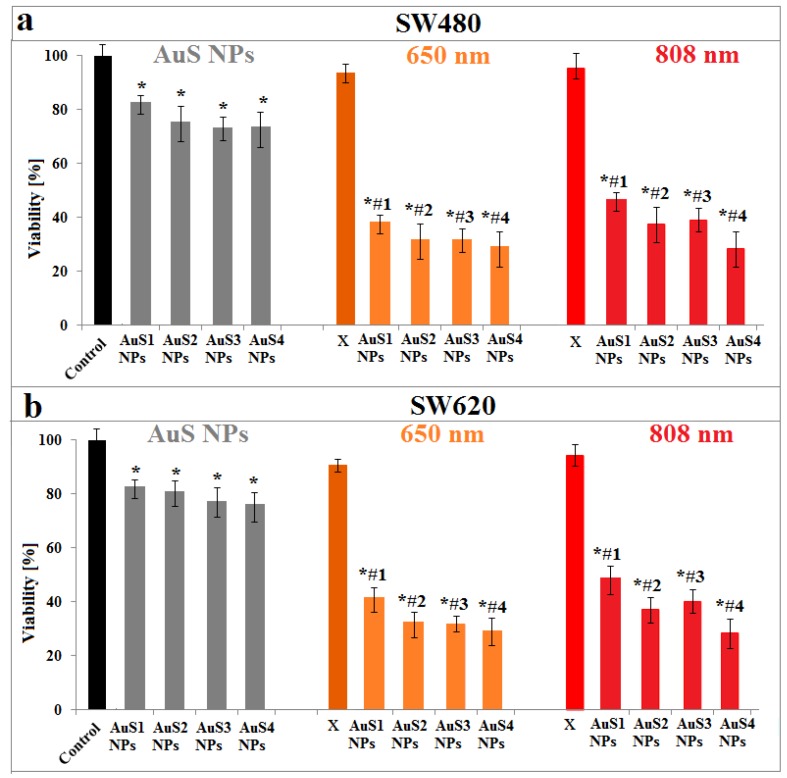
Viability of colon cancer SW480 (**a**) and SW620 (**b**) cells: after addition of AuS1–AuS4 NPs and nonirradiated (grey columns) as well as irradiated by 650-nm (orange columns) and 808-nm (red columns) lasers: Data was considered as significant when * *p* < 0.05 vs. Control; # *p* < 0.05 vs. AuS1–AuS4 NPs. “X” corresponds to cells cultured without AuS NPs and irradiated for 5 min.

After synthesis and characterization of the obtained AuS NPs, we investigated the possibility of using them in PTT of cancer. For this purpose, we cultured colon cancer cells with AuS1–AuS4 NPs and, next, we irradiated these cells with 650-nm and 808-nm lasers for 5 min. To evaluate the effectiveness of simulated PTT in the presence of AuS NPs, light microscopy images and MTS assay were performed; see Figure 7 and Figure 8, respectively.

Light microscopy images of colon cancer cells (Figure 7) allowed to observe the changes in cell morphology. Moreover, changes in the number of dead cells caused by the addition of AuS NPs as well as by the laser irradiation in the presence of gold nanostars were visible when we compare these samples with control cancer cells cultured without AuS NPs. The smallest changes in morphology as well as in the number of cells were noticed in the samples cultured without AuS NPs, however, when irradiated by lasers for 5 min in comparison with control SW480 and SW620 cells. The reason for the occurrence of morphological changes in the cells cultured with AuS NPs is the fancy shape of the nanoparticles, which provide a large active surface. Especially, the star arms are the locations responsible for the active surface enlargement. It is noted that nanoparticles with large active surfaces are more toxic than for example spherical nanoparticles [16,17]. This high toxicity is correlated with the large number of active sites, which can interact with cancer cells, generating free radicals and oxidative stress [27,29]. These kinds of surfaces are also responsible for the optical and photodynamic properties of nanoparticles [30]. Therefore, nanoparticles with the largest values of parameter *a* (see Table 1) should have the most effective photothermal properties. Consequently, in our study, the most visible changes in the morphology and number of dead cells are visible for samples irradiated by lasers and cultured with AuS4 NPs having the longest arms.

The changes in the viability of cells cultured with AuS NPs, nonirradiated and irradiated, as well as cultured without AuS1–AuS4 NPs and irradiated by lasers, were determined using the MTS assay; see Figure 8.

MTS assay showed a decrease of viability of cancer cells irradiated with 650- and 808-nm lasers in comparison with controls. However, this decrease is not significant in contrast with the values of viability of cells cultured with AuS NPs. The mortality of SW480 cells cultured with gold nanostars is between 18% for AuS1 NPs and 27% for AuS4 NPs, while for SW620 cells, they are between 18% and 24%. The arms of AuS4 NPs were the longest, which is visible in the TEM images, Figure 1, and in Table 1. In gold nanostars, the surface activity mainly depends on the star arms lengths. The main contact surface with the cells are the star arms, causing changes in the cells and leading to cell death [31,32]. However, the toxicity effect of AuS NPs depends also on the concentration of gold nanostars [33]. Therefore, in our experiment, we used the same concentration of AuS1–AuS4 NPs; thus, we can assume that the length of star arms is responsible for the differences in cells death. When we compare the viability of cells cultured with AuS NPs non irradiated with the ones irradiated by lasers, we can say that the nanoparticles themselves are not toxic at the used concentration. For SW480 cells cultured with AuS NPs, the viability of cells is between 26% and 38% and between 24% and 43% for 650-nm and 808-nm lasers, respectively. For SW620 cells, the decreases of cell viability caused by culture with AuS NPs and irradiation by lasers are between 74% and 60% and between 76% and 53% for the respective wavelengths. Interestingly, also in the case of irradiated cells, the highest values of cell mortality are observed for the AuS4 NPs, while the smallest are for the Au1 NPs with the shortest arms, Table 1. The multiple sharp arms of AuS NPs act like “antennas”, which can convert the electron energy from wavelengths to heat energy. Liu et al. synthesized 30-nm and 60-nm gold nanostars as well as spherical Au NPs, and they compared the photothermal conversion efficiency of these three kinds of gold nanoparticles. The results showed that the value of photothermal conversion efficiency for gold nanospheres is 60% of the η values obtained for gold nanostars [34]. Furthermore, they showed a 10 °C increase of temperature in AuS NP solutions after irradiation. In our study, we also observed an increase of temperature close to 10 °C; see Figure 6. Moreover, they showed that the natural construction of blood vessels in cancer cells caused the nanoparticles to be accumulated in and around cancer cells. Therefore, it is possible to reduce the laser power needed to precisely destroy cancer cells when we culture these cells with gold nanostars [35]. Moreover, the results obtained from the MTS assay correlated nicely with the results obtained from light microscopy images; see Figure 7.

## 3. Materials and Methods

### 3.1. Materials

CTAB, HAuCl_4_, silver nitrate (AgNO_3_), sodium borohydride (NaBH_4_), ascorbic acid (C_6_H_8_O_6_), and all other chemicals were ordered from Sigma–Aldrich (Saint Louis, Missouri, USA).

### 3.2. Synthesis of Au Nanostars with Different Lengths of Star Arms

First, solutions of CTAB and HAuCl_4_ were prepared as follows: 0.364 g of CTAB was dissolved in 5 mL of H_2_O, while 0.0017 g HAuCl_4_ was dissolved in 10 mL of H_2_O. Next, 5 mL of HAuCl_4_ solution was added to the CTAB solution and mixed at 30 °C under vigorous stirring. After dissolving the solution, 0.6 mL of 100 × 10^−3^ M NaBH_4_ was added. When the solution color changed to red, the reaction was stopped, and in this way, solution A was obtained. Next, 0.364 g of CTAB was dissolved in 5 mL of H_2_O under vigorous stirring, and 0.2 mL of 3.97 × 10^−3^ M of AgNO_3_, 5 mL of 5 × 10^−4^ M of HAuCl_4_, 140 μL of 7.9 × 10^−2^ M C_6_H_8_O_6_, and 30 μL of solution A were added to the CTAB solution and mixed under vigorous stirring to obtain a blue color. To investigate the sizes of star arms, the reactions were performed using two, three, and four times higher reducer concentrations

### 3.3. TEM Characterization

The morphology of the synthesized Au nanostars was examined by scanning transmission electron microscopy (STEM – Titan Thermo Scientific) using the bright field detector (BF) in conventional mode. Selected area electron diffraction (SAED) patterns were taken in the TEM mode. All these measurements were performed on an aberration-corrected FEI Titan electron microscope operating at 300 kV equipped with an FEG cathode. The particle size distribution was evaluated based on high-resolution scanning transmission electron microscopy (HRSTEM) images taken from different areas of the TEM grids. For each sample, the diameters of 100 nanoparticles were measured.

### 3.4. X-Ray Diffraction

Microstructural studies of the synthesized nanoparticles were performed by X-ray diffraction (XRD) analysis using a two-circle laboratory diffractometer Panalytical X’Pert Pro (Malvern Panalytical Ltd) with Cu K_α_ X-ray source operating at 40 kV and 30 mA. Microstructural parameters such as lattice constants and average coherent scattering length were refined with the Rietvield method through the Fullprof software [36]. The same software was used for diffractogram fitting, where a modified Thompson–Cox–Hastings pseudo-Voigt function was used as a profile function and where the background was fitted by a 6-coefficient polynomial function.

### 3.5. UV-Vis Spectroscopy

Lambda Bio20 instrument from Perkin Elmer was used to measure the UV-Vis spectra of the AuS NPs. The scan speed was 240 nm/min, while the resolution was 1 nm. In this experiment, the used spectral range was from 200 nm to 900 nm.

### 3.6. Cell Culture

Colon cancer cell lines (SW480 and SW620) were obtained due to courtesy of Prof. Caroline Dive, Paterson Institute for Cancer Research, University of Manchester. These cell lines were cultured in DMEM with high glucose (Corning, NY, USA) in a 37 °C humidified atmosphere with 5% CO_2_. All media were supplemented with 10% fetal bovine serum (FBS, Biowest, Nuaille, France) and gentamicin (50 µg/mL) (PAN-Biotech, Aidenbach, Germany). The cells were cultured by biweekly passages and were regularly tested for *Mycoplasma* sp. contamination by PCR-ELISA kit (Roche, Mannheim, Germany) according to the manufacturers’ instruction.

### 3.7. Optical Microscopy Images of Cells

The images of control cells as well as of cells cultured with and without AuS NPs with 3.88 × 10^−3^ mg/mL concentration and irradiated and nonirradiated by lasers were taken at 100× magnification using an optical microscope Olympus IX70 (Olympus Corporation, Tokyo, Japan).

### 3.8. MTS Assay

Cytotoxic activity of AuS NPs against human colon cancer cells (SW480 and SW620) was determined using 3-(4,5-dimethylthiazol-2-yl)-5-(3-carboxymethoxyphenyl)-2-(4-sulfophenyl)-2H-tetrazolium (MTS) assay (CellTiter 96^®^ AQueous One Solution Cell Proliferation Assay, Promega, Madison, WI, USA). Briefly, cells were cultured in flat-bottom 96-well plates (Sarstedt, Numbrecht, Germany) at a density of 1 × 10^4^/well in DMEM medium containing 10% FBS. After 24 h, 20 μL of 3.88 × 10^−3^ mg/mL Au NPs solution was added to the cells. After additional 24 h of culture, 20 µL of MTS (CellTiter 96^®^ AQueous One Solution Cell Proliferation Assay, Promega) dye solution was added per well and incubated for 2 h. The quantity of formazan product, directly proportional to the number of living cells in culture, was detected by absorbance measurements at 490 nm with a 96-well plate reader (Spark^®^ Tecan, Mannedorf, Switzerland).

### 3.9. Photothermal Conversion Efficiency Determined from the Time Constant

For photothermal properties measurements, solutions of AuS NPs were placed into glass cuvettes and irradiated with low-intensity LED arrays created with 650- and 808-nm wavelengths lasers, respectively. A digital multimeter connected to a small Pt-100 thermo-resistor located inside of the cuvette was used to measure the temperature evolution during irradiation. The temperature evolution during laser irradiation can be described by the following equation:

(1) $$\eta =\frac{\left(c_{w}m_{w+}c_{n}m_{n}\right)\Delta T}{IA\Delta t}$$

where *cw* and *cn* are the specific heats of water and gold, respectively; *mw* and *mn* are the masses of water and gold, respectively; Δ*T* is the temperature increase in AuS NP solutions in the Δ*t* time interval, *A* is the illumination area of the fluid in the experiment, and *I* is the laser incident power. In this experiment, we used 3.88 × 10^−3^ mg/mL concentrations of AuS NPs.

### 3.10. Light Source and Cells Irradiation Protocols

The SW480 and SW620 colon cancer cells cultured with 3.88 × 10^−3^ mg/mL of AuS1–AuS4 NPs were irradiated using low-intensity lasers operating at two different wavelengths: 650 nm and 808 nm. The irradiation time was 5 min, while the intensity of the array was 100 mW/cm^2^. We used this time because, after that period, differences in the viability of cells were not observed.

### 3.11. Statistical Analysis

The results obtained from the MTS assay are represented as the means ± SEM (the standard error of the mean) with the T test. *p* value < 0.05 was considered statistically significant.

## 4. Conclusions

In this paper, the possibility of using the AuS NPs in simulated PTT in cancer cell lines was investigated. Therefore, as it was confirmed by TEM and XRD, we synthesized crystalline gold nanostars with similar values of core diagonals (between 140 nm and 146 nm), however, with different sizes of star arms lengths: from 193 nm for AuS1 NPs to 356 nm for AuS4 NPs. The different lengths of star arms were obtained during the synthesis by controlling the reducer concentration. Moreover, UV-Vis spectroscopy showed that the position of SPR peak depends on the width of the end of the star arm, and it is shifted from 640 nm for AuS1 NPs with the narrowest end to 770 nm for AuS4 NPs with the widest end. Thus, the photothermal efficiency of the obtained AuS NPs depends on the width of the end of star arm. The highest increase of temperature as well as the highest values of photothermal efficiency are calculated to be in AuS4 NPs, with the widest end of star arm. Effective photothermal properties of AuS NPs we determined by irradiating the samples with 650-nm and 808-nm lasers. For this purpose, the viability and changes in the morphology of two SW480 and SW620 colon cell lines cultured with AuS1–AuS4 NPs and irradiated by 650-nm and 808-nm lasers were investigated. For both cells, the viability of cells was between 24% to 43% and was dependent on the used AuS NPs and laser wavelengths while the viability of cells cultured without AuS NPs and irradiated was around 90%. These results showed that AuS NPs can find potential application in photothermal cancer therapy. Moreover, in this paper, we also showed that the photothermal properties of AuS NPs depend on the widths of the end of star arm and the length of the arm itself and that they are increasing with the enlargement of the end width and elongation of the star arms, which is correlated with the increase of reducer concentration.

## Figures and Tables

**Table 1:** Parameters for the four different sets of AuS NPs.

Parameter (nm)	Samples			
	AuS1 NPs	AuS2 NPs	AuS3 NPs	AuS4 NPs
**a**	193.75 ± 17.11	243.75 ± 15.21	293.75 ± 16.76	356.27 ± 19.25
**b**	28.13 ± 4.21	19.38 ± 3.17	26.25 ± 4.58	44.38 ± 6.32
**c**	3.75 ± 1.02	5.63 ± 2.12	5.00 ± 1.87	6.25 ± 2.09
**b/c**	7.50 ± 2.28	3.44 ± 2.75	5.25 ± 3.01	7.10 ± 3.02
**d**	146.38 ± 4.27	143.75 ± 3.77	140.63 ± 5.08	146.21 ± 5.27

The colors correspond to the respective samples and the colors in the first row on the left correspond to the respective parameters of the nanostars shown in Figure 1.

**Table 2:** Values of the lattice parameters of the fabricated AuS NPs.

**Lattice Parameter (Å)**	**Samples**			
	**AuS1 NPs**	**AuS2 NPs**	**AuS3 NPs**	**AuS4 NPs**
	**4.0740 (6)**	**4.0763 (3)**	**4.077 (1)**	**4.0784 (2)**

The colors correspond to the respective samples.

**Table 3:** Values of photothermal conversion efficiencies (ratio of the internal energy increase of the fluid to the total incoming radiation input) for the obtained AuS NPs irradiated by 650-nm and 808-nm lasers. The background colors correspond to the respective samples.

*η* (%)	Samples			
	AuS1 NPs	AuS2 NPs	AuS3 NPs	AuS4 NPs
***η_650_***	**38**	**40**	**39**	**44**
***η_808_***	**33**	**32**	**33**	**34**
